# Effects of Primary Mast Cell Disease on Hemostasis and Erythropoiesis

**DOI:** 10.3390/ijms22168960

**Published:** 2021-08-20

**Authors:** Holger Seidel, Hans-Jörg Hertfelder, Johannes Oldenburg, Johannes P. Kruppenbacher, Lawrence B. Afrin, Gerhard J. Molderings

**Affiliations:** 1 Center for Bleeding Disorders and Transfusion Medicine (CBT), Am Propsthof 3, D-53121 Bonn, Germany; h.seidel@cbtmed.de (H.S.); h.hertfelder@cbtmed.de (H.-J.H.); jk@cbtmed.de (J.P.K.); 2 Institute of Experimental Haematology and Transfusion Medicine, University Hospital Bonn, Venusberg-Campus 1, D-53127 Bonn, Germany; Johannes.Oldenburg@ukbonn.de; 3 Department of Mast Cell Studies, AIM Center for Personalized Medicine, 3010 Westchester Ave Suite 404, Purchase, NY 10577, USA; drafrin@aimcenterpm.com; 4 Institute of Human Genetics, University Hospital of Bonn, Venusberg-Campus 1, D-53127 Bonn, Germany

**Keywords:** mast cell disease, hemostasis, erythropoiesis, bleeding, fibrinolysis, heparin

## Abstract

Mast cell disease is an epigenetically and genetically determined disease entity with very diverse clinical manifestations in potentially every system and tissue due to inap pro priate release of variable subsets of mast cell mediators together with accumulation of either morphologically normal or altered mast cells. Easy bruising, excessive bleeding, and aberrancies of erythropoiesis can frequently be observed in patients with mast cell disease. A thorough history, including a family history, will guide the appropriate work-up, and laboratory evaluations may provide clues to diagnosis. In recent years, our understanding of the involvement of coagulation and anticoagulant pathways, the fibrinolytic system, and erythropoiesis in the pathophysiology of mast cell disease has increased considerably. This review summarizes current knowledge of the impact of the disturbed hemostatic and erythropoietic balance in patients with mast cell disease and describes options of treatment.

## 1. Introduction

Mast cells (MCs) are hematopoietic tissue immune cells that act both as effector and regulatory cells (e.g., [1]) in adaptive and innate immunity (e.g., [2]). This versatility is reflected in the myriad of immunologic and non-immune activation stimuli (e.g., by G protein-coupled receptors) resulting in the secretion of a large number (>1000) of pre-stored mediators (e.g., histamine, tryptase) and numerous de novo-synthesized lipid mediators (e.g., eicosanoids), chemokines, and cytokines [3].

Primary MC disease comprises a group of historically defined different disease entities (consisting of several variants; Table 1): systemic mastocytosis (SM), MC activation syndrome (MCAS), cutaneous mastocytosis (CM), MC sarcoma and hereditary alpha-tryptasemia (HAT). SM, CM, and MC sarcoma are rare. The prevalence of MCAS, at least in Germany, is about 17% [4] and about 20% in the U.S. [5], and that of HAT was found to be 4–6% of the general population [6], hence both being common disorders. Two genome-wide association studies (GWAS; [7,8]) on patients with SM revealed different non-overlapping results with regard to the multiple mutations in a variety of genes. A recent GWAS on MCAS patients detected also a large number of SNPs without overlapping with the GWAS results in SM patients (Hänisch 2021, personal communication; manuscript in preparation). A next generation sequencing study also revealed a myriad of germline mutations in a large number of genes [9]. In addition, in qPCR studies on >35 genes in SM as well as MCAS patients, a multitude of somatic mutations have been detected ([9]; further references therein). In synopsis, these data suggest a new view of the development and categorization of primary MC disease in that it is one polygenic multifactorial disease entity characterized by epigenetic and presumably consequently genetic alterations (somatic and germline mutations) in a variety of genes. The combinatorial calculated number of possible combinations of the genetic alterations suggests that each patient affected by a primary MC disease has a unique mutational pattern or profile driving a unique pattern of aberrant MC mediator production and release. This inappropriate release (both constitutively and reactively) of variable subsets of MC mediators, together with accumulation of either morphologically ordinary mast cells due to impaired apoptosis (MCAS and well-differentiated SM) [1] or morphologically altered and immunohistochemically identifiable mutated mast cells (SM and MC leukemia), can affect single or multiple systems (though multisystem presentations are far more common), usually manifesting with symptoms in a subacute or chronic waxing/waning or recurrent manner ([10], further references therein). Due to both the widespread distribution of MCs in the organism and the great heterogeneity of aberrant mediator expression patterns, symptoms can involve virtually all organs and tissues; thus, the clinical presentation of primary mast cell disease is very diverse (Table 1 in [1]). Opposite effects can appear in different patients (e.g., polycythemia [11] versus red cell aplasia [12]), or at different times in an individual patient (e.g., alternating diarrhea and constipation (e.g., [13]), or even in different sites at the same time in an individual patient (e.g., co-existing osteoporosis and osteosclerosis (e.g., [14,15])). Severity of symptoms, too, in one tissue/organ/system can vary substantially from one patient to the next, at different times in an individual patient, or even in different systems at the same time in an individual patient.

In the present review we summarize the impact of primary MC disease on blood coagulation and erythropoiesis.

## 2. Impact on Hemostasis and Thrombosis

**Figure 1:**
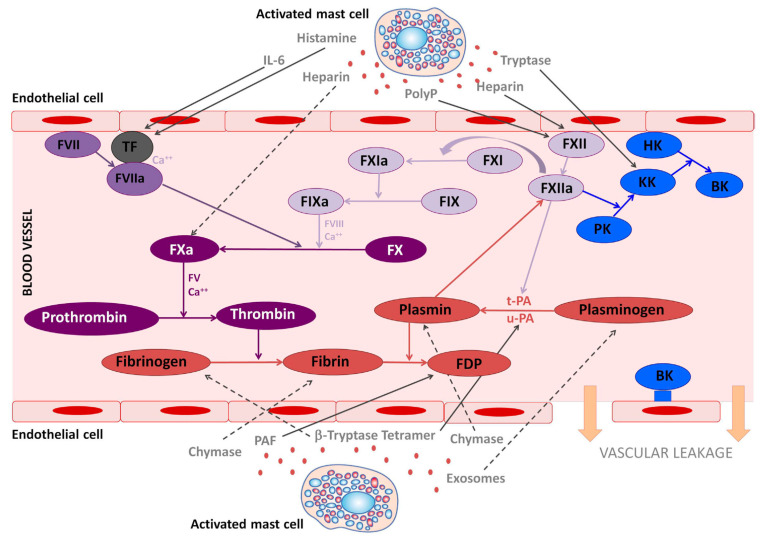
The involvement of mast cell mediators in the coagulation and kallikrein–kinin system. The figure illustrates the effects that mast cells mediators released upon activation during anaphylaxis exert in the kallikrein–kinin, coagulation, and fibrinolytic systems. Solid lines represent activated pathways. Dashed lines are inhibitory pathways. Kinin-forming system factors are represented in blue; the fibrinolytic system is represented in red; the common coagulation pathway in dark purple; the extrinsic coagulation pathway in medium purple; the intrinsic coagulation pathway in light purple. PolyP, polyphosphates; TF, tissue factor; PK, prekallikrein; KK, Kallikrein; BK, bradykinin; HK, high molecular-weight kininogen; tPA, tissue plasminogen activator; uPA, urokinase plasminogen activator; FDP, fibrin degradation products; PAF, platelet-activating factor. (Legend and figure adapted from [16]).

MC activation may affect hemostasis by vascular (e.g., endothelial cells) and cellular components (e.g., platelets, monocytes and neutrophils) as well as clotting and fibrinolytic factors (Figure 1; [16]). The array of substances released by activated MCs which may influence hemostasis includes histamine, heparin, vasoactive intestinal polypeptide (VIP), prostanoids, the proteases tryptase and chymase, tissue-type plasminogen activator (tPA), factor VIII, etc. Since high concentrations in circulation and other tissues of these mediators can be achieved by their release from MCs, both clinically significant bleeding and thrombosis can occur in MC disease ([17]; further references therein). Platelet activation may trigger MC activation, and hence stimulate MC mediator release, contributing to distortion of hemostasis [18].

### 2.1. Mast Cells as Parts of Bleeding Diatheses

Clinical signs of a bleeding diathesis, such as hematoma formation, bruising, prolonged bleeding after biopsies, gingival bleeding, epistaxis, gastrointestinal hemorrhage, conjunctival hemorrhage, menorrhagia or hemorrhagic ulcer disease occur in about 50% of patients with MC disease ([7,19]; further references therein) and can contribute to deterioration in quality of life. Thus, bleeding diathesis represents a frequent and clinically relevant problem in MC disease, although severe or fatal bleeding seems to be rare [20,21,22,23]. The presence of hemorrhagic disorders in patients with MC disease is mainly explained by the anticoagulant activity of MC degranulation products like heparin, histamine and tryptase [24] and by hyperfibrinolysis [17]. In human tissues, MCs are the main source of heparin [25,26]. Increased release of heparin from MCs can reach circulating concentrations similar to heparin levels achieved during thrombo prophylaxis by subcuta-neously applied heparin [17,27] and, thereby, could contribute to the bleeding diathesis. As MC activation can be quite a focal (i.e., non-systemic) process or event, and since many of the MC mediators (including many with pro- or anti-coagulant activity) are very thermolabile and have very short half-lives in vivo, detecting laboratory evidence of aberrant coagulation resulting from MC activation sometimes can be quite difficult even with clinically remarkable presentations, e.g., extensive bruising with little to no abnormalities in routine coagulation system testing, including von Willebrand factor testing. Careful attention to specimen handling (especially continuous chilling), and repeat testing, are required not uncommonly. The anticoagulant effect of heparin consists of binding to antithrombin leading to inactivation of thrombin and factor Xa. Under these conditions, thrombin generation, as measured by formation of thrombin–antithrombin complex and prothrombin fragment F1 + 2 levels, is inhibited. In addition, in systemic MC disease, acquired von Willebrand syndrome (AVWS) has been described [28,29]. However, the validity of explanation that heparin binding to von Willebrand factor (VWF) causes VWF dysfunction as the cause of AVWS has not been demonstrated convincingly: heparin use for anticoagulation has not yet been related to VWF dysfunction and AVWS. Thus, another, yet unidentified mechanism must impair platelet adhesion and aggregation ([28,29]; further references therein) to cause AVWS.

In MC disease, endothelial cell activation is triggered by bradykinin, which is released by proteolytic cleavage of high molecular weight kininogen by kallikrein ([30]; Figure 1). Additionally, bradykinin is a potent stimulator of tissue-type plasminogen activator release from endothelial cells. Endothelial cells and MCs represent an important source of tissue-type plasminogen activator (tPA) [31], indicating that MC activation plays an important role in endogenous fibrinolysis. Thus, it could be hypothesized that these profibrinolytic effects of MC activation physiologically may be preventive for venous thrombosis [32], which is supported by a MC-deficient mouse model [33]. The release of tPA from endothelial cells might also lead to an early destabilization of hemostatic clots by fibrinolysis. Studies to date suggest that the main causes of bleeding diatheses in primary MC disease likely include pathological hyperfibrinolysis in MCAD with elevation of tPA levels released in significant amounts also from MCs and endothelial cells as well without concomitant release of plasminogen activator inhibitor type 1 (PAI) [31], decrease of PAI activity and significantly increased plasminogen activation indicated by increased plasmin–antiplasmin (PAP) complexes [17], and increased expression of urokinase-type plasminogen activator (uPA; encoded by the gene PLAU; [34]).

The important MC mediator histamine can influence hemostasis by upregulating thrombomodulin activity in endothelial cells and thereby promoting the activation of the protein C/S (PC/S) system by thrombomodulin-bound thrombin [35]. The PC/S system is a major anticoagulant that is required for the downregulation of blood coagulation. Moreover, histamine also stimulates endothelial cells to release VWF [36] and tPA [37]. Another MC mediator, prostaglandin D2, is known to inhibit platelet aggregation via activation of platelet adenylate cyclase ([38]; for review, see [39]).

MC tryptase could have opposite effects on hemostasis as both an anticoagulant and a procoagulant (see below). The antithrombotic function is mainly due to the ability of tryptase to proteolytically degrade fibrinogen, predominantly by cleavage of the C-terminal α-chains before thrombin can convert fibrinogen to fibrin and subsequently impair fibrin polymerization ([24,40]; further references therein). Tryptase also acts directly on the fibrinolytic pathway by activating the uPA resulting in the direct, i.e., fibrin-independent conversion of plasminogen into plasmin. Therefore, uPA promotes the proteolytic degradation of fibrinogen and other clotting factors.

Finally, thrombocytopenia caused by hypersplenism ([41]; further references therein) and vitamin K deficiency due to MC mediator-induced malabsorption might enhance bleeding.

In this context, it can be speculated whether the intracerebral bleeding which occurs in about 0.6% of patients with Long Covid syndrome [42] might be linked to dysfunctional MCs in patients with MC disease [43]. Meanwhile it has been revealed that COVID-19 disease is predominantly a vascular disease, leading to leaky blood vessels [44] which outlasts the acute infection. In the presence of certain non-identified intracellular distortions in dysfunctional MCs, a MC mediator-related bleeding may appear by diapedesis. We are currently treating a patient with SM, in whom 6 months after a moderate COVID-19 disease, the first brain bleeding appeared by diapedesis. Two other such bleedings followed at intervals of about 4 months (unpublished observations). Interestingly, the three bleeding events were preceded hours to a few days by bleeding into the sclera or the vitreous body of the eye. All bleeding responded well to treatment with 4 × 500 to 3 × 1000 mg of tranexamic acid (TXA, depending on the intensity of the central nervous symptoms).

In all patients suspected of having a MC disease, a MC disease-specific examination of the coagulation system, where necessary expanded to include investigations for possible simultaneous genetic thrombophilia-promoting mutations such as factor V Leiden and prothrombin mutation G20210A (since primary MC disease is an epigenetic disorder, genetically determined comorbidities could also result from this epigenetic disturbance), should be carried out for diagnostic reasons [27] and before surgeries to plan the perioperative procedures [45].

#### 2.1.1. Laboratory Diagnostics

Since MCs represent the main source of endogenous heparin [46], it is important to try to determine endogenous heparin and its release by MCs when feasible. However, determination of heparin levels by chromogenic anti-Factor Xa (anti Xa) assay is challenging for several pre-analytic reasons. In contrast to drug-derived heparin, endogenous heparin from MC release is less stable, as platelet activation neutralizes endogenous heparin by the release of platelet factor 4, leading to lower or non-measurable anti-Factor Xa levels. Therefore, tubes containing citrate, theophylline, adenosine, and dipyridamole (CTAD) should be used for heparin stabilization to avoid the neutralization of heparin in the pre-analytic phase after blood sampling [47]. The rapid degradation and neutralization of heparin by platelet activation at ambient temperature can be minimized additionally by handling the blood specimen on ice or in a refrigerated environment (approx. +4 °C) and by centrifugation within 15–30 min after phlebotomy. On the other hand, falsely high anti-Xa levels might be measured if CTAD tubes were contaminated with heparin during their manufacturing process. Thus, newly applied batches of CTAD tubes should be tested for measurable heparin (anti Xa) levels before using them to avoid the detection of artificially high heparin levels.

At first, baseline heparin (anti-Xa) levels should be sampled. For proving MC-mediated release of heparin, as a provocation test venous occlusion of the upper arm for 10 minutes (venous occlusion test, VOT) can be performed, using a blood pressure cuff inflated 10 mm Hg above diastolic pressure. This standardized mild non-pharmacological test might be able to stimulate MC activation and degranulation by hypoxia and increased compartment pressure [17,27,48,49]. The measured anti-Xa levels can reach similar amounts achieved during thromboprophylaxis by subcutaneously applied heparin but are often beyond it. In rare cases, mostly attributed to MC leukemia, very high heparin levels might be determined and may be responsible for severe bleeding. MCAS-diagnosing clinicians should be aware that low or non-measurable anti-Xa levels do not exclude a MC disease because of the above-mentioned challenges. Elevated heparin levels (baseline and after VOT) can be observed in 59% in patients with MCAS and SM [27]. Therefore, evidence of increased plasma heparin increases the likelihood of the presence of a MC disease but is not in itself definitive diagnostic proof. Additionally, as for all MC mediators, there is no correlation between anti-Xa levels and severity or number of MC-associated symptoms, but anti-Xa levels are more sensitive than other mediators for detecting MCAS [27].

Hyperfibrinolysis in MCAS patients with bleeding diatheses can be identified by increased levels of tPA and of plasmin–antiplasmin complexes (PAP) [17] using the above mentioned VOT. Activation of hemostasis may be reflected by increased levels of D-dimer. Apart from D-dimer, profibrinolytic parameters such as PAP, tPA, and PAI, as well, are not routinely measured parameters. Thus, their determination is limited to specialized laboratories. Since mild bleeding tendencies can reinforce each other additively, other hemorrhagic disorders, e.g., Von Willebrand’s disease, should be excluded. A frequent finding in investigation of coagulation deficiency is deficiency of one or more of the vitamin K-dependent clotting factors, which is usually attributed to diet and malabsorption. However, vitamin K deficiency is rarely causally related to bleeding in MC disease. The impact of rather ‘crude’ coagulation tests such as the group tests of ‘clotting time’, ‘thrombin time’, thromboelastography (a rather more the whole blood coagulation recording mechanical system than sensitive platelet function detecting system) in MC disease is low (or “poor”). Additionally, clotting factor XIII here in general is not relevantly altered. In addition, plasminogen, a component with high plasmatic concentration is poorly influenced by MCs but plasmin activation is indicated by the generation of PAP complexes and correlates well with the release of tPA. PAI 1 may also be altered by tPA resulting in decline of level due to formation of tPA–PAI complexes. D-dimer levels depend on the presence of fibrin. They appear to be highly variable according to individual patients and do not directly correlate to MC disease activity. Platelet dysfunction may also occur in some patients with MC disease and concomitant disease such as Ehlers Danlos syndrome but appear to be intrinsically related to MC disease itself.

#### 2.1.2. Treatment Options in Mast Cell Mediator-Induced Bleeding

In the absence of placebo-controlled trials, hemostatic treatment of mild bleeding in MC disease patients is based only on clinical evidence. Before and after surgeries, MC activity should be reduced as much as possible by medications and other maneuvers (e.g., avoidance of triggers, such as temperature shock from a cold operating room or infusion of refrigerated fluids) ideally determined at an earlier, non-emergent point to be ideal for this task. Of note, though systemic medications may be sufficient, sometimes topical medications may be needed, or at least more helpful, for controlling bleeding in topically accessible sites (e.g., a nasal spray of an H_1_ blocker or cromolyn for epistaxis, or a vaginal douche or suppository of such drugs for menorrhagia [50]). Routine procedures for arresting surgical bleeding rarely are effective when that bleeding is induced by MC mediators [20], so alternative approaches often are needed. Unless contraindicated, tranexamic acid (TXA) 1 g should be administered intravenously shortly before the first incision, and, depending on the intra- and postoperative bleeding situation, TXA infusion should be continued for, at least, 12–24 h (total dosage 2–3 g/24 h). In the case of severe thrombocytopenia, transfusion of platelet concentrates should be considered.

Antifibrinolytic drugs such as TXA or epsilon aminocaproic acid (EACA) often are effective due to several mechanisms: 

(I) One of the major bleeding signs in patients with MC disease is mucocutaneous bleeding from local hyperfibrinolysis in well-vascularized tissues, e.g., endometrium, bladder, gums, or regions of the ear, nose and throat.

(II) Antifibrinolytics target endogenous fibrinolysis, which is involved in increased pathologically irritable MC activation. The synthetic lysine-analogue TXA and also EACA competitively block the binding of plasminogen to fibrin via its lysine-binding sites and, therefore, inhibit the tPA-mediated activation of plasminogen to plasmin [51]. The binding of TXA to plasminogen is 6–10 times more potent than that of EACA [52].

(III) Given the link between MC-mediated inflammatory response and increased fibrinolysis, TXA feasibly attenuates the inflammatory response. This possible anti-inflammatory effect of TXA has been demonstrated in randomized controlled trials in cardiac surgery, measuring biomarkers (IL-6, fibrin degradation products, plasminogen activator inhibitor) and cardiac markers (CK, troponin I), respectively [53,54].

(IV) Finally, TXA may inhibit the complement system, as has been observed in patients with hereditary angioneurotic edema [55], e.g., by normalization of plasma kinin activation. In this context, it is of note that TXA is approved for allergic conditions like urticarial swelling in Japan [56].

Depending on the clinical circumstances, TXA can be administrated topically, orally, or intravenously. The risk of arterial or venous thromboembolism using TXA remains unclear but appears to be low with the dosages usually applied. TXA should be administrated with caution in patients with renal impairment to treat bleeding of the urinary tract because the ureter does not provide tPA release intraluminally. Thus, in the case of urinary tract bleeding, the fibrinolysis of clots might be irreversibly blocked by TXA leading to ureter and/or urethral obstruction.

In MC disease patients with vitamin K deficiency, vitamin K could be applied orally in high doses (5–10 mg p.o.) or intravenously in case of disturbed absorption. Thus, vitamin K substitution should be considered in (malnourished) patients with severe bleeding symptoms and/or patients undergoing surgery with a high bleeding risk.

In case of very high plasma heparin levels and clinically overt bleeding, the application of protamine chloride to neutralize heparin should be considered. Protamine chloride is derived from salmon melt and is used to reverse anticoagulation with unfractionated heparin (UFH) and—less effectively—low-molecular-weight-heparin (LMWH). However, adverse reactions to protamine, such as hypotension, might be related to the release of inflammatory mediators, including histamine, by MCs. Therefore, protamine use should be limited to MC disease patients with life-threatening bleeding in presence of measurable endogenous heparin. In these situations, heparin could be neutralized by protamine titration to the patient’s heparin levels. In rare cases of adverse protamine effects, administration of recombinant activated factor VII (rFVIIa) might be used as an alternative in case of life-threatening bleeding. Apart from the possibility of bleeding, in patients with MC disease, LMWH, UFH or fondaparinux as thromboprophylaxis should not be avoided when thromboprophylaxis is required because of internal or surgical treatment.

### 2.2. Mast Cells as Parts of Increased Thrombophilia

In parallel with inducing a bleeding diathesis, MCs are thought to contribute to venous thrombo embolism and atherosclerosis [57,58] through release of granular constituents including histamine, prostanoids [59], heparin (by its activation of factor XII (FXII)), cytokines, the proteases tryptase and chymase (via activation of protease-activated receptors and clotting factors such as fibrinogen, FXII, and XIII), platelet activating factor (PAF, which activates platelets and leads to fibrin formation via Factor XII activation), secretion of VWF, Factor VIII [60] (which is also present in MCs [61,62]), soluble P-selectin, and increased intercellular adhesion molecule-1 (ICAM-1) expression. FXII activation by heparin and/or anionic polyphosphates initiates the intrinsic pathway of coagulation. The MC proteases tryptase and chymase bind electrostatically with exceptionally high affinity to heparin, increasing their stability and, hence, protecting them from inhibition by physiological inhibitors, thus promoting their catalytic properties. Both proteases degrade the alpha-, beta- and gamma-chains of fibrinogen, and these degradation products prolong thrombin-induced clotting time of human plasma [63]. Thus, these proteases may initially act prothrombotic and then, later on, antithrombotic. Beyond these mediator effects, too, activated MCs expose the inorganic polymer polyphosphate (polyP) on their surfaces, which initiates procoagulant and proinflammatory reactions [64,65]. MCs also release extracellular traps, and the presence of MC-derived traps has been reported in coronary thrombi [66,67]. Similar to neutrophil extracellular traps, MC extracellular traps might stimulate thrombosis. Whether altered availability of its molecular constituents predisposes the above-described fibrinolytic repair system toward thrombophilia remains to be determined. Interestingly, findings indicate that MC-released histamine may either cause or inhibit thrombosis, depending on whether it acts on resting endothelial cells or on cells pre-activated by other inflammatory stimuli [68]. It also fits with Brown et al. (2013) [68] that MCs, by producing t-PA in a resting state and by expressing PAI-1 when activated by C5a complement, switched from a profibrinolytic to a prothrombotic phenotype [69]. Of course, for thrombophilic events in MC disease, possible underlying distribution of thrombophilic risk factors in the general population should be kept in mind, too.

#### Therapeutic Procedures in Patients with Mast Cell Disease and Thrombophilia

For reducing the risk of thrombosis and bleeding in patients with primary MC disease in their everyday lives, MC activation should be reduced as much as possible by the profile of medication found best in the individual patient. Given the extreme combinatorial epigenetic and genetic complexity of primary MC disease, resulting in extreme interindividual heterogeneity in aberrant constitutive and reactive mediator release and thus extreme heterogeneity in clinical presentation, it is unsurprising that the optimal treatment profile is unique to the individual patient, and it also is unsurprising that a very large array of treatments has been found helpful in various MC disease patients [70]. Of note, the H_1_ histamine receptor antagonist rupatadine may theoretically be the best choice of such antagonists for MC disease patients with thrombotic tendencies because it also inhibits PAF.

## 3. Impact on Erythropoiesis

Abnormalities in quantity or function of any of the molecules (many of which can be found in the large repertoire of mast cell mediators) directly or indirectly affecting any of the many steps in the erythropoietic (and red cell degradation/recycling) processes have potential for driving not only abnormal quantities (high or low) of circulating erythrocytes but also abnormal erythrocytic qualities. (Table 2).

As one could accurately say about the development of any cell population in eukaryotic organisms, normal erythropoiesis is the result of an exquisitely choreographed ballet of a great many distinct molecules, in the case of erythropoiesis resulting in production of not only normal structured individual erythrocytes (with normal membranes encasing normal amounts of normally structured hemoglobin) but also normal numbers of such erythrocytes (roughly 2.4 million per second, or about 200 billion per day), which then are released from the marrow into circulation at rates appropriate to the prevailing physiologic circumstances in the body—and later (on average a bit more than three months after emergence from the marrow) are normally decomposed (with subsequent recycling of component molecules).

Given the huge menagerie of mediators produced and released by the MC, and with most MC mediators naturally driving a wide range of direct and indirect, local and remote effects, the range of symptoms which potentially can be produced by chronic aberrant MC activation is vast. Yet, three general themes become apparent on study of the matter: inflammation, allergic-type phenomena, and dystrophisms (aberrancies in growth and development in potentially any tissue)—and though various MC disease patients may have highly varying extents of allergic and dystrophic issues, chronic multisystem inflammation clearly is the “universal constant” of MC disease. Depending on degree and chronicity, inflammation usually drives relative or even absolute anemia (the latter seen in about two-thirds of MCAS patients [10]). However, as judged by their erythrocyte counts and hemoglobin and hematocrit levels vis-à-vis the clinically obvious extent of their inflammation, many MCAS patients enjoy surprisingly robust erythropoiesis, producing a relative or even absolute polycythemia (the latter in about 8% of MCAS patients [10], usually mild and non-progressive, easily distinguishing it from untreated polycythemia vera), suggesting expression by their dysfunctional MCs of either mediators directly or indirectly antagonizing the arrays of mediators normally expressed in inflammation and/or mediators directly or indirectly stimulating erythropoiesis. Clinicians evaluating MC disease patients should recognize that a “normal” red cell count and hemoglobin and hematocrit levels in the setting of chronic significant inflammation are not normal and represent a pro-erythropoietic manifestation of the disease. Low-dose imatinib (typically, 200 mg daily) has been found helpful in polycythemic MCAS patients (e.g., [11]). Polycythemia in MCAS, however, of course is not necessarily due to MC disease (i.e., a diagnosis of MC disease does not render the patient immune from developing other problems), and other causes (e.g., familial polycythemias, polycythemia vera, hypoxemia of any cause, erythropoietin-secreting tumors (e.g., liver and kidney tumors, hemangioblastomas), occult erythropoietin administration, excessive overt or occult/inadvertent testosterone use (e.g., flaxseed oil)) need to be excluded, usually a fairly straightforward process based on sufficiently detailed history, physical examination, and a modicum of testing.

Although palpable splenomegaly is not uncommon in systemic mastocytosis, it is uncommon to find splenomegaly detectable by either palpation or imaging in MCAS patients (even in spite of 19% of MCAS patients reporting chronic intermittent left upper quadrant tenderness, likely reflecting MC activation-driven splenitis). As such, sequestration is rarely a mechanism of anemia in MCAS. Hemolysis, too, appears to be uncommonly driven by MC disease, though of course MC disease does not prevent other hemolytic ailments from emerging independently of a patient’s MC disease. MC disease seems to have some propensity to spur the humoral immune system to errantly/inappropriately produce a wide variety of antibodies (unreported observations, author LBA), and as such antibodies usually are randomly targeted, most probably are not detectable by assays for specific antibodies, and many of the detected antibodies clearly (by aberrant titer patterns over time and absence of associated clinical disease) are not “on-target” antibodies reflecting the true presence of infectious or autoimmune diseases—but occasionally such an errantly produced antibody has sufficient specificity to drive a clinically apparent autoimmunity, such as an autoimmune hemolytic anemia, an anti-phospholipid antibody syndrome (driving thrombosis or bleeding), or autoimmune rheumatologic, endocrinologic, or neurologic diseases. Standard treatments for such diseases are needed, but concomitant control of the MC activation may yield even better outcomes (as is sometimes seen in the setting of SM with associated hematologic neoplasms (SM-AHN) and in MCAS-associated cancers, too (e.g., [71])).

Although inflammation-driven anemia (usually normocytic and normochromic) is expected and common in MC disease, other (MC activation-driven and non-MC activation-driven) causes of anemia can also be present and should be suspected and further evaluated when red cell indices seem inappropriate for an inflammation-driven anemia vis-à-vis the extent of clinically apparent inflammation. With regard to microcytic anemias (seen in 24% of MCAS patients [10]), iron deficiency is common and, usually, identified easily on testing. All routine differential diagnostic thinking as to the causes of a detected iron deficiency need to be considered, and it needs to be recognized, too, that the chronic multisystem inflammation of MC disease often includes duodenitis which can lead to selective micronutrient malabsorption syndromes including iron malabsorption and even copper malabsorption. Abnormalities in iron indices in MC disease patients can be misleading. Abnormal (more often low than high) levels of iron, iron saturation, and/or ferritin (sometimes even contradictory levels) are seen not uncommonly in MC disease, but diagnosis (let alone treatment) of iron deficiency should be approached cautiously if classic microcytic hypochromic anemia itself is not present. In other words, given that the marrow is the body’s largest iron consumer by far, absence of microcytosis, hypochromia, and anemia despite iron parameters suggestive of iron deficiency is actually more suggestive of a situation in which, despite the obvious presence of abnormalities of various sorts in the body’s management of its iron absorption, storage, and transport, the marrow nevertheless is continuing to access all the iron needed to manufacture all the hemoglobin needed to adequately stock the 200 billion normally sized erythrocytes normally produced each day. Treatment of iron deficiency also needs to be approached cautiously in MC disease patients from the perspective that many such patients adversely react (likely precisely because of their dysfunctional MCs) to such treatments, both oral and parenteral, and MC activation-targeted pretreatment (e.g., H_1_ ± H_2_ histamine receptor antagonists) not uncommonly is needed prior to parenteral iron treatments. In addition, intravenously applied iron is stored in the reticuloendothelial system for long periods of time. Ergo, if iron is activating a MC disease patient’s dysfunctional MCs, this activation will persist for a long period. MC disease patients often react to medication product excipients, so a patient’s failure to tolerate one iron oral or parenteral iron supplement does not ensure intolerance of other supplements mixed with different excipients. Organic oral iron polypeptide formulations sometimes are better tolerated than inorganic iron salts (e.g., sulfates, gluconates). Occasionally, appropriately matched transfusion of packed washed leukocyte-poor red cells is the only way to tolerably address iron deficiency (one unit of packed red cells contains all the iron the body needs for a year at normal consumption rates). In this context, however, although transfusion therapies usually are well tolerated in MC disease patients, one must also consider that transfusion therapies may elicit immunological effects which are able to activate MCs, thereby aggravating MC disease.

Moreover, therapy-refractory anemia may be induced by MC disease-induced kidney disorders ([72,73]; further references therein), a microenvironment in the bone marrow disturbed by pathological MCs ([74]; further references therein), or myelofibrosis due to certain MC mediators (e.g., [75]). Recently, too, it has been reported that erythro cytes damaged oxidatively by inflammatory processes demonstrated erythro phagocytosis by activated MCs [76].

Sometimes, specific proximate causes of anemia or polycythemia can be identified, but then the typical roots of those proximate causes cannot be found (or, less commonly, are initially mistakenly thought to be present), in which case the possibility that MC activation may be the underlying issue needs to be considered. For example, idiopathic pure red cell aplasia (PRCA) has been reported in association with MCAD ([12,77]; further references therein) and was found in at least one case [12] to be refractory to standard treatments for PRCA but then responded well to MCAS-targeted treatments including H_1_ and H_2_ histamine receptor antagonists as well as low-dose imatinib at one point and oral cromolyn at another point, this last response intriguingly suggesting (given the near-complete absence of absorption of oral cromolyn, and given the drug’s short half-life) that mediators inappropriately released specifically from proximal luminal gastrointestinal tract MCs were (directly or indirectly) intensively inhibiting erythropoiesis. In another unreported example highlighting the diversity of MC mediators and their effects, author LBA identified an MCAS patient also suffering both sickle cell anemia and dialysis-dependent end-stage kidney disease resulting in unusually severe anemia occasioning transfusion dependence despite high-intensity erythropoietin treatment—and in whom treatment with montelukast for chronic mild asthma quickly resolved the excess anemia and the transfusion dependence. Additionally, in yet another example [11] emphasizing the potentially polar opposite effects of aberrant mast cell mediator expression on erythropoiesis in different patients, idiopathic polycythemia was misdiagnosed as polycythemia vera and failed to respond to therapeutic phlebotomy, but then, when MCAS was found in the patient, responded quickly and well (both in symptoms and polycythemia) to low-dose imatinib.

Macrocytosis (typically mild and stable) is common in MCAS (about 29% of such patients [10]), and though specific causes (e.g., cobalamin or folate deficiency, hemolysis, etc.) must be sought, they are found only rarely. Rather, most macrocytosis in MCAS seems more likely due to premature release of reticulocytes, though the specific aberrant mediator expression patterns driving such marrow behavior are unclear.

Since MCAS is common, most aberrant erythropoiesis in primary MC disease (or MC disease in general) is due to aberrant MC activation. However, a tiny fraction of the total MC disease population suffers substantial MC neoplasia, and though aberrant MC activation in some fashion, to some extent, is “part and parcel” of every neoplastic MC disorder, it must be remembered, too, that high marrow tumor burdens observed in some types of MC disease (e.g., SM) of course can further contribute to anemia from simply physically compromising the amount of space in the marrow available for erythropoiesis. In such cases, therapies which are able to reduce MC numbers in the bone marrow (e.g., some kinase inhibitors, interferon α, or certain cytotoxic agents) are indicated.

## 4. Conclusions

Disturbances of hemostasis and erythropoiesis often occur in primary MC disease and may or may not be consequential to the MC disease. Specific cause(s) of any given such disturbance should be sought and specifically treated, which may require MC-specific or non-MC-specific interventions. Prophylactic interventions may be warranted in certain (e.g., peri-operative) circumstances.

## Figures and Tables

**Table 1:** Classification of primary mast cell disease.

Primary Mast Cell Disease				
Systemic Mastocytosis (SM)	Mast Cell Activation Syndrome (MCAS)	Cutaneous Mastocytosis (CM)	Mast Cell Sarcoma	Hereditary Hypertryptasemia
Clinical variants				
Indolent SM Well-differentiated SM Smoldering SM Aggressive SM SM with an associated hematological neoplasm Mast cell leukemia	Irritable bowel syndrome phenotype Fibromyalgia phenotype Cardiac phenotype CNS-phenotype Idiopathic anaphylaxis phenotype Mixed phenotype Others	Maculopapular CM = urticaria pigmentosa Diffuse CM Solitary cutaneous mastocytoma		

**Table 2:** Impacts of mast cell disease on quantities and qualities of erythrocytes.

Affected Erythrocytic Parameter	Type/Direction of Aberrancy	Subtype/Mechanism/Comments
Quantity	Erythrocytosis/polycythemia	Polycythemia driven by chronic aberrant mast cell expression of erythropoietic mediators (e.g., erythropoietin, activin A, etc.) is not uncommon; rarely, male MCAS patients may drive polycythemia through androgen use to treat chronic fatigue; erythropoietin-secreting tumors induced by mast cell activation are rare.
		Polycythemia vera is rare, but mast cell disease of any type does confer increased risk for any type of acute and chronic hematologic malignancies.
	Erythropenia/anemia	Anemia of chronic inflammation is common.
		Anemia of iron deficiency driven by insufficient iron intake (due to dietary and medication intolerances) or insufficient iron absorption (due to disease-driven or iatrogenic gastric acid insufficiency and/or duodenal inflammation) or bleeding (see below) is common.
		Anemia of copper deficiency due to disease-driven or iatrogenic gastric acid insufficiency is uncommon.
		Chronic kidney disease (and related anemia) is common, but the proportion of such disease driven by mast cell disease (dominantly MCAS) has not yet been identified.
		Gastrointestinal tract bleeding (due to mast cell activation-driven ulcer disease or vascular malformations, occasionally even tumors) is not uncommon.
		Genitourinary tract bleeding (principally menorrhagia due to aberrant heparin release and fibrinolysis from dysfunctional endometrial mast cells) is very common in MCAS.
		Anemia due to marrow compromise by high tumor load is common in the rare disease of systemic mastocytosis.
		Sequestration (e.g., hepatosplenomegaly) is rare.
Quality	Macrocytic	Mild macrocytosis is common in MCAS and systemic mastocytosis, likely from premature release from marrow of maturing erythrocytes due to aberrant mast cell mediator expression; hemolysis, sequestration, massive splenomegaly, cobalamin or folate deficiency, or hematologic malignancy are seen rarely or uncommonly.
	Microcytic	Mild to moderate microcytosis is common in MCAS, most likely from iron deficiency, rarely also from copper deficiency.
	Dyspoietic	Ineffective erythropoiesis (as in myelodysplastic syndrome) is uncommon.
	Hemolytic	Hemolysis (autoimmune or non-immune, congenital or acquired, acute or chronic) is rare.

## Data Availability

Not applicable.
